# Emotional Mimicry in Social Context: The Case of Disgust and Pride

**DOI:** 10.3389/fpsyg.2012.00475

**Published:** 2012-11-02

**Authors:** Agneta H. Fischer, Daniela Becker, Lotte Veenstra

**Affiliations:** ^1^Department of Social Psychology, University of AmsterdamAmsterdam, Netherlands

**Keywords:** emotional mimicry, facial mimicry, disgust, pride, affiliation, social context

## Abstract

A recent review on facial mimicry concludes that emotional mimicry is less ubiquitous than has been suggested, and only occurs in interactions that are potentially affiliative (see Hess and Fischer, in revision). We hypothesize that individuals do not mimic facial expressions that can be perceived as offensive, such as disgust, and mimic positive emotion displays, but only when the context is affiliative (i.e., with intimates). Second, we expect that in spontaneous interactions not mimicry, but empathic feelings with the other predict the accurateness of emotion recognition. Data were collected in a pseudo-experimental setting, during an event organized for subscribers of a large Dutch women’s magazine. One woman (expresser) was exposed to two emotional stimuli (i.e., a vile smell, a compliment) in order to evoke disgust and pride respectively. Another woman (observer: intimate or stranger) was sitting opposite of her. We collected self-report measures on emotions and empathy, and coded facial expressions of disgust and smiling on the basis of FACS. The results show that participants do not mimic disgust. In contrast, smiles displayed after the vile smell and the compliment were mimicked, but only among intimates. We also found that self-reported empathy and not mimicry is related to the recognition of disgust. These findings are discussed in the light of a Social Contextual view on emotional mimicry.

## Introduction

How do we react to someone showing faces of fear, disgust, or happiness? One answer to this question is that we mimic these expressions and show similar faces of fear, disgust, and happiness, respectively. The automatic mimicry of non-verbal emotion expressions (Hatfield et al., 1994; Dimberg et al., 2000) is assumed to have two important social functions, namely fostering social bonds, and helping to understand and empathize with others’ emotions. However, a recent review of the literature Hess and Fischer (in revision) has raised questions about the consensus that the prevailing response to an emotional facial reaction is mimicry. They argue that the definition of emotional mimicry is unclear and that the evidence, in particular for the mimicry of negative emotions, is rather limited.

This lack of consistent evidence can be explained from a Social Contextual perspective (e.g., Keltner and Haidt, 1999; Fischer et al., 2003; Parkinson et al., 2005; Fischer and Manstead, 2008; Van Kleef, 2009; Parkinson, 2011; Hess and Fischer, in revision). A Social Contextual view holds that mimicry serves a social function, and is dependent on the social context in which the emotion is expressed. Emotional mimicry is the imitation of an emotional intention rather than the movement of facial muscles and we only mimic if the emotional signal and the relationship are perceived as affiliative, and if we want to affiliate. Indeed, there are many situations in which mimicry of negative emotions and even positive emotions could be non-affiliative, and thus rather dysfunctional from a social point of view, for example when our partner is angry at us, or when our friend shows fear for a small spider, or when our enemy laughs at us.

Whereas social context effects on mimicry have been investigated in previous research (e.g., McHugo et al., 1985, 1991; Lanzetta and Englis, 1989; Hess et al., 1999; Yabar and Hess, 2007; Likowski et al., 2008; Van der Schalk et al., 2011), evidence from experimental contexts where the interaction partner is actually present is scarce. However, we think this type of evidence is important for studying social functions of mimicry, because in actual interactions the social effects of emotional mimicry are expected to have more impact than when one is watching a non-respondent target on a photo or in a video. In the present study we test hypotheses following from a Social Contextual view on emotional mimicry. We evoke two emotions, disgust and pride, and examine whether observers mimic these emotions to the same extent among intimates and strangers, and whether the recognition of disgust and pride is determined by attempts to empathize and/or by mimicking.

### Evidence for emotional mimicry

Many studies have addressed facial mimicry (e.g., Dimberg, 1982; Dimberg and Lundqvist, 1990; Lundqvist, 1995; Lundqvist and Dimberg, 1995; Dimberg and Thunberg, 1998; Dimberg et al., 2002), leading to a general consensus that there is abundant evidence that we mimic each other’s emotions. However, Hess and Fischer (in revision) concluded that the empirical evidence for the existence of emotional mimicry is limited. First, in the majority of studies only two emotions have been included, namely anger and happiness, and the occurrence of mainly smiling and frowning in reaction to these two displays have been regarded as indicative of facial mimicry. Second, studies that have included more than two emotions have predominantly shown that we frown more in reaction to angry, fearful, sad, or disgust faces than to neutral faces (e.g., Lundqvist and Dimberg, 1995; Hess and Blairy, 2001; Magnée et al., 2007; Bourgeois and Hess, 2008; Likowski et al., 2008; Weyers et al., 2009).

However, frowning is a rather a-specific facial reaction, and these findings therefore need not necessarily reflect mimicry. A frown basically signals that something is wrong and thus needs our attention (Kaiser and Wehrle, 2001), and may indicate various negative emotions, as well as a negative mood, concentration, concern, or effort. More conclusive evidence for the mimicry of discrete emotions should show facial movements of similar muscles as the one’s displayed, such as the Frontalis for surprise (lifting the eyebrows), or the Levator Labii Alaeque Nasi for disgust (nose wrinkling). Studies including these facial muscles have produced inconsistent effects, however. In the case of disgust, for example, mimicry effects measured with activity of the Levator were found in only one of three studies (Lundqvist and Dimberg, 1995). Hess and Fischer (in revision) therefore concluded that empirical data on facial mimicry to date mainly justify the conclusion that we react to emotional faces with facial displays that are similar in valence, but not that we mimic discrete emotions, such as anger, disgust, sadness, or fear. We may explain this lack of evidence when considering the social functions of mimicry.

### Social functions of emotional mimicry

The occurrence and functions of emotional mimicry can be explained from a Social Contextual perspective. Following other social functional accounts of contagion and empathy (e.g., Hatfield et al., 1994; Keltner and Haidt, 1999; Parkinson, 2005, 2011; Fischer and Manstead, 2008; Hess and Fischer, in revision), we argue that emotional mimicry serves an affiliation function and thus should only occur when the emotional signal can be perceived as affiliative and when mimicry would promote social bonds and mutual understanding.

This implies first of all that mimicry depends on the relationship between expresser and observer, because the interpretation of an emotional signal also depends on how one relates to the expresser. The importance of this relationship is indeed evident from behavioral mimicry studies (Cheng and Chartrand, 2003; Lakin and Chartrand, 2003; Lakin et al., 2003; Stel and Vonk, 2010), but also from emotional mimicry studies. For example, a more positive attitude toward the target (Likowski et al., 2008), or a more cooperative social context (Lanzetta and Englis, 1989), or the target being an ingroup member have all shown to elicit more mimicry (Hess et al., 1999; Yabar and Hess, 2007; Van der Schalk et al., 2011). These results thus suggest that we mimic the emotions of similar others, friends or intimates more than the emotional displays of dissimilar others, strangers or competitors.

Second, these and other studies also demonstrate that mimicry not only depends on the relational context, but also on the nature of the emotional display, for example whether it signals approach or rather attack, or distancing. In examining the spontaneous mimicry of smiles and frowns in different public settings, for example, Hinsz and Tomhave (1991) found that many people mimic strangers’ smiles, but they hardly mimic strangers’ frowns.

We therefore hypothesize that negative emotions that signal attack or criticism, such as anger or disgust (Roseman et al., 1994) will generally not be mimicked, because they do not intrinsically signal empathy or understanding. Although previous research has sometimes found congruent displays in reaction to anger or disgust, we expect that in an actual interaction the presence of an interaction partner is more salient, which may reduce or even dissolve mimicry in reaction to an offensive display. This is nicely illustrated in a study by Bourgeois and Hess (2008), who found that participants do not mimic anger, but they do mimic the smiles of a fellow student when narrating an anger experience. In addition, social effects may also be more salient if one actually knows the other person well and may be amplified with the intimacy of the relationship. Indeed, Häfner and IJzerman (2011) showed that individuals high in communal strength mimic their partner’s anger less than individual slow in communal strength.

Whereas these studies have focused on anger, the same may apply to disgust, as recent research (Chapman et al., 2009) has shown that disgust expressions can also be evoked in reaction to offensive stimuli in the social domain. Therefore, disgust faces, like anger faces can also be interpreted as directed at the observer, or at least interpreted as unpleasant. A social context in which disgust is expressed can thus often be considered non-affiliative.

The display of positive emotions, in contrast, is generally an affiliative signal, reflecting rapport, understanding, or solidarity (see e.g., Bourgeois and Hess, 2008). However, this is only the case to the extent that the relationship is potentially affiliative (see also Stel et al., 2010). Thus, whereas it is most likely that we mimic the smiles of our friends and intimates, and would even mimic the smiles of strangers in a neutral, potentially affiliative situation (e.g., as a signal of politeness or recognition or shared amusement), we would not mimic a smile that could be interpreted as hostile, or offensive, and thus as non-affiliative. This could occur, for example, when strangers smile when they clearly feel Schadenfreude, or inappropriate amusement in response to a racist joke. In the same vein, the proud smile of a stranger would generally not be mimicked, as this expression may imply that the other thinks of him or herself as better, thereby increasing social distance (Markus and Kitayama, 1991). On the other hand, friends may share their pride, because they share the recognition of each other’s achievement (i.e., basking in reflected glory), rendering it more likely that pride smiles would be mimicked (e.g., Fischer and Manstead, 2008). In addition, smiles of friends who share an amusing, or even awkward situation would also be mimicked more than smiles of strangers in similar situations (see also Fridlund, 1991; Hess et al., 1995; Jakobs et al., 1999, 2001). In other words, smiles that are perceived to signal affiliation and shared feelings are generally more likely to be spontaneously mimicked, which is the case when friends or intimates smile to each other.

A second function that has been proposed for emotion mimicry is the facilitation of the recognition of others’ emotions (Lipps, 1907). The basic assumption is that observers mimic emotion expressions, which generates facial feedback, which in turn helps the observer to recognize the other’s non-verbal display through emotional simulation (e.g., Niedenthal, 2007). The relation between mimicry and emotion recognition has been examined in various types of studies, but provided inconclusive evidence (see Parkinson, 2011; Hess and Fischer, in revision). In one line of research the function of mimicry has been examined by comparing two conditions in which participants’ mimicry is either blocked or not (e.g., Blairy et al., 1999; Niedenthal et al., 2000; Stel and van Knippenberg, 2008; Hawk et al., 2011; Maringer et al., 2011). The blocking of specific facial muscles affected the speed of recognition of emotions, either in a positive or negative way, depending on the task and judgment context. Other studies, however, did not find support for the role of mimicry in emotion recognition. For example, Hess and Blairy (2001) measured participants’ facial displays and self-reported emotions when decoding short videos of natural facial expressions. They did not find evidence for the idea that either self-reported emotions or emotion mimicry predicted accuracy in emotion recognition. In line with previous theorizing and research (e.g., Hatfield et al., 1994; Hess and Blairy, 2001; Bogart and Matsumoto, 2010; Parkinson, 2011), and because we think mimicry is less prevalent as often assumed, we think that mimicry is not a necessary condition for correctly interpreting another’s emotion.

In addition, various studies have shown that emotion expressions may not only elicit mimicry, but also feelings of empathy (e.g., Sonnby-Borgström, 2002; Sonnby-Borgström et al., 2003; Lamm et al., 2008) or perspective taking (Hawk et al., 2011). Hawk et al. (2011) for example, show that observing an embarrassed emotion display of a person dancing on a silly song, elicits both mimicry and perspective taking, as measured by self-reports. We may therefore suggest that an empathic stance could also help to understand or correctly interpret another’s emotion (see also Preston and de Waal, 2003). Especially when mimicry is absent because of potential negative effects, the empathy felt by the observer may be more crucial in correctly identifying others’ feelings.

### The present study

In the present study we test hypotheses following from a Social Contextual view on emotional mimicry. We evoke two emotions, disgust and pride. We chose disgust because it is an emotion that is easily evoked and previous studies on its neural underpinnings have shown that both seeing and experiencing disgust activates similar areas in the brain, which would make mimicry of the disgust expression maximally likely (Wicker et al., 2003). Moreover, disgust has a very clear and specific set of facial movements, namely the Nose Wrinkler (AU9), the Upper Lip Raiser (AU10), and AU43 (closing the eyes). If observers mimic disgust, there should be a significant correlation between these facial movements of the observer and expresser. In addition to these specific disgust expressions, we also examined the frown (AU4) as a more general index of negative mood or a signal of worry, and smiles (AU12), as an index of affiliation, appeasement, or amusement.

We chose pride as a positive emotion, because pride is a discrete emotion that has recently been shown to have unique facial expressions (Tracy and Robins, 2004; Mortillaro et al., 2012). It differs from happiness in that it clearly is not an emotion that would be shared unconditionally with others, because pride is triumph about one’s own achievements, and therefore need not elicit pride in others, unless those others are intimates or friends. Pride is therefore an interesting emotion to test the social functions of smile mimicry.

In sum, our *first* prediction is that individuals do not mimic disgust, because disgust is a non-affiliative signal, and the mimicry of disgust is therefore not socially functional. *Second*, we predict that individuals mimic positive expressions (i.e., smiles) both during the disgust event, as well as during the pride event, but only when the relationship between expresser and observer is affiliative (e.g., Chartrand and Bargh, 1999; Lakin et al., 2003; Stel et al., 2010). *Third*, we predict that observers’ empathy, and not their facial mimicry, will mediate the relationship between the expresser’s disgust and pride, and the recognition of these emotions.

## Materials and Methods

### Participants and design

We recruited 278 persons during a Dutch summer festival (“Libelle Zomerweek”). We first deleted six couples that included men, because we expected gender differences in emotional expressiveness, and there were too few men to be able to compare the results for men and women. In the original experiment we also had a condition (*N* = 120) in which the expresser had to hide her emotions. We do not report this condition in the present paper. This leaves 146 women. Because the quality of the videos on the first day was bad (too dark), we had to leave out the data of 34 other women. This leaves us with 112 women (*M*_age_ = 45.31, SD_age_ = 12.65; 96.4% Dutch), in 56 couples. Participants within each couple were either intimates (friends or family, *N* = 60) or strangers (*N* = 52). The assignment to either condition was however not completely random. Those participants who were together with a familiar person were coupled and assigned to the intimates condition, while those participants who approached us as single visitors were randomly coupled with another, unfamiliar, person, and assigned to the strangers condition. In some occasions, we also asked two couples of intimates to split up and rearrange as two couples of strangers. Additionally, within each couple participants were assigned to either be the expresser, or the receiver on the basis of the chair they chose. They could not know which chair was selected for the expresser and which for the observer. Participation was entirely voluntary.

### Procedure

After having provided informed consent, participants were instructed to enter a cubicle together with their partner (intimate vs. stranger) and the experimenter. In the cubicle, the experimenter asked the two participants to choose one of the two available chairs. Depending on their choice of the chair, they were either the expresser or the observer. Both were introduced to the experiment and were told that in the next 5 min one of them had to complete small and simple tasks, while the other person would just observe. They then received a written instruction to “behave naturally during the whole experiment”. Expressers were additionally asked to “freely show their feelings and thoughts to their partner”, while receivers were instructed to “simply focus their attention on their partner.” The experimenter stressed that they were not allowed to speak during the experiment, but that they should stay in (eye) contact.

To evoke disgust, the experimenter asked the expresser to evaluate the smell of two “brand new” cleaning agents. While one of the non-transparent flasks was filled with conventional fabric softener, the other flask was filled with a strong water solution of asafetida, an Eastern spice with a pungent, unpleasant smell (also known as “devil’s dung”). After listening to the product evaluation cover story, expressers opened the first bottle, always containing the conventional fabric softener, and smelled it. Then they closed the first bottle, opened the second flask, containing asafetida, and smelled it. Then the experimenter asked which one of the two they preferred.

After this first event, two other emotions were evoked (surprise, disappointment), which will not be reported in the present paper. The last emotion that was elicited was pride. We had participants look at a booklet containing four photos of crying babies. We told them that one of these babies was a boy and they had to figure out which one was the boy. In order to elicit pride, the experimenter introduced the task by telling them that this was a very difficult task and that many people made mistakes. They then could look at the photos for however long they wanted. When they gave their answer, the experimenter always said that this was the correct answer and praised them for being very clever (independent of which answer they gave). After the last part of the experimental session, both participants left the cubicle and were administered a questionnaire about the experiment.

### Measures

We had two slightly different questionnaires for the expresser and observer. First of all, the expressers were asked to report the intensity of several *emotions* after the smelling of the second flask, and after receiving the compliment, whereas the observers were asked what they thought their partner felt during these events. Both the expresser and the observer could rate the intensity of nine different emotions, seven negative (irritation, disgust, sadness, surprise, shame, sadness, and stress) and two positive (happiness and pride). They were asked to indicate the intensity with which they had felt, or thought their partner had felt, each of these emotions. For all items an 8-point Likert scale was used, ranging from 0 (*not at all*) to 7 (*very strong*).

To measure the degree of *empathy* between the partners, expressers and observers were asked to indicate the extent to which they felt empathy with the other. For the observers, the items were as follows: “I shared my partner’s emotions,” “I empathized with my partner,” and “I saw that my partner felt the same emotion as I did,” “I felt a strong bond with my partner,” Cronbach’s alpha_observers_ = 0.71; Cronbach’s alpha_expressers_ = 0.80). In order to check whether intimates and strangers indeed differed in the nature of their relationship, we measured the amount of *familiarity* persons felt for the other (“To what extent do you feel familiar with the other person?”). At the end, we asked for their age, nationality, and education. After having completed all self-report measures, participants were debriefed and dismissed.

### Facial expressions

In order to code the facial expressions of both the expresser and the receiver during the task, we recorded each participant on video camera throughout the experiment (using two cameras who filmed the participants from a frontal perspective). We selected the video fragments for the disgust expressions, starting with the moment that the expressers started smelling at the second bottle with the disgusting smell until they put it down. On average the fragments were about 5 s long. The video fragments for the pride event were selected immediately after the experimenter gave the compliment to the expresser and before they started talking about the expresser’s answer. These fragments took about 2 s on average.

The first author coded the intensity of the following action units on the basis of Ekman’s Facial Action Coding System (1978). For disgust: AU9 (Nose Wrinkler), AU10 (Upper Lip Raiser), AU43 (closing the eyes^1^), AU4 (Brow Lowerer, or frown), and AU12 (Lip Corner Puller, or smile). AU9 and AU10 were coded as one expression, which we will refer to as AU9/10, because it was very hard to see the distinction between the two facial movements, partly because many of the women wore glasses (see also Hawk et al., 2012, for a similar decision). For pride, one action unit was scored: AU12 (smile). A score of 0 indicated that the action unit was not present, a score of 3 indicated that the action unit was very strongly visible.

In order to calculate inter reliability of the coding, a certified FACS coder coded 61% of the participants (*N* = 68). Correlations between the two coders were high and significant (all *p*’s < 0.0001, for AU9.90; for AU4.91, for AU43.80, and for AU12.84).

## Results

### Checks

We first examined whether the emotion of disgust and pride were adequately evoked. With regard to disgust, a repeated measures ANOVA with the expressers’ self-reported disgust, stress, irritation, and surprise right after smelling the disgusting smell (the other emotions were almost always rated as “*not at all*”), showed a significant effect, *F*(3, 53) = 115.738, *p* < 0.0001, ${\text{η}}_{p}^{2}=$ 0.87. Disgust was reported as much more intense (*M* = 5.00, SD = 2.12) than was surprise (*M* = 0.80, SD = 2.00), irritation (*M* = 0.35, SD = 1.25), or stress (*M* = 0.02, SD = 0.13). A Paired-Samples *t*-test comparing disgust and the averaged scores on stress, irritation, and surprise showed that disgust was reported as more intense [*t*(55) = 14.31, *p* < 0.0001].

With respect to pride, a repeated measures ANOVA with the expressers’ self-reported pride, happiness, disappointment, and surprise felt after the compliment also showed a significant effect, *F*(2, 54) = 57.10, *p* < 0.0001, ${\text{η}}_{p}^{2}=$ 0.36. Pride was reported as most intense (*M* = 3.78, SD = 2.61), followed by happiness (*M* = 2.39, SD = 2.88), surprise (*M* = 1.11, SD = 2.20), and disappointment (*M* = 0.02, SD = 0.13). A Paired-Samples *t*-test comparing pride and the averaged scores on happiness, disappointment, and surprise showed that pride was reported as more intense [*t*(55) = 6.50, *p* < 0.0001]. Even the comparison with only happiness, proved to be significant, [*t*(55) = 2.55, *p* = 0.014]. Thus, although pride was felt as less intense than was disgust, the elicitation of both emotions can be considered successful.

We then checked whether these feelings of disgust and pride in the expresser were correlated with expresser’s facial movements that are typically associated with these emotions. We found significant positive correlations between expresser’s self-reported feelings of disgust and looking away (AU43; *r* = 0.32, *p* = 0.018), but not with nose wrinkling (AU9/10, *r* = 0.20, *p* = 0.15). We also did not find a positive correlation between feelings of disgust and frowning (AU4; *r* = 0.22, *p* = 0.11) The three Action Units were positively correlated with each other: AU9/10 (nose wrinkling) with AU4 (frowning, *r* = 0.62, *p* < 0.0001), and with AU43 (looking away, *r* = 0.56, *p* < 0.0001), and AU 4 with AU43 (*r* = 0.56, *p* < 0.0001). In the case of pride we did not find significant correlations between the self-reported feeling of pride or happiness, and smiling (all *p*’s > 0.40). The absence of significant correlations between pride or happiness and smiling is not completely unexpected, because smiling has a variety of meanings (see also Niedenthal et al., 2010). We will discuss this further in the Discussion section.

Finally, we checked whether participants in the intimate condition felt more familiar with each other than the participants in the stranger condition. This was indeed the case, showing that friends (*M* = 6.25, SD = 0.96) felt more familiar than strangers (*M* = 1.44, SD = 1.10; *F*(1, 104) = 571.19, *p* < 0.0001, ${\text{η}}_{p}^{2}=$ 0.85)^2^. In addition, friend observers (*M* = 5.44, SD = 0.97) also felt more empathy with the other than stranger observers (*M* = 4.78, SD = 1.00), *F*(1, 52) = 5.83 *p* = 0.019, ${\text{η}}_{p}^{2}=$ 0.10.

### Emotional mimicry in reaction to disgust

In order to examine disgust mimicry, we computed correlations between the negative facial displays of observers and expressers (see Table 1). No significant correlations were found, confirming our prediction that observers did not mimic disgust. We then split the file for intimates and strangers, and used the composite score of AU9, AU43, and AU4, in order to have more observations per cell. No significant correlation was found, either for intimates (*r* = −0.04, *p* = 0.85), or for strangers (*r* = 0.16, *p* = 0.43). As expected, however, correlations of expressers’ and observers’ smiles during the disgust event were significant in the intimate condition (*r* = 0.47, *p* = 0.009), but not in the stranger condition (*r* = −0.05, *p* = 0.82).

**Table 1:** **Correlations between facial actions of expresser and observer after the disgust stimulus**.

Observer	AU9/10(*nose wrinkling*)	AU 43 (*closing eyes*) + *looking away*	AU4 (*frowning*)	AU12(*smiling*)
EXPRESSER				
AU9/10	0.04	0.18	0.12	0.23#
AU43 (+ *looking away*)	0.03	−0.02	0.13	0.03
AU4	−0.05	0.02	−0.12	0.05
AU12	−0.02	0.06	0.08	0.19

**Note*. #*p* = 0.08*.

We then examined the amount of disgust expressiveness as a function of expressers versus observers and intimates versus strangers. We conducted a repeated measures ANOVA with nature of relation (intimates, strangers) as between-subjects factor, expresser and observer as within-subject factor, and the scores of AU9, AU43, and AU4 as dependent variables. A main effect of expresser-observer was found, *F*(3, 52) = 10.83, *p* < 0.0001, ${\text{η}}_{p}^{2}=$ 0.38, showing that expressers displayed a greater intensity of all facial expressions (see Table 2), but no main effect, nor interaction with the nature of the relationship was found. In other words, intimates did not express more disgust than strangers, but observers clearly showed less disgust than expressers. We also conducted a repeated measures ANOVA with smiling as dependent measure, and nature of relation as between-subjects factor, and again found no effect of the nature of the relation, and no difference between the intensity of smiling of expressers and observers (see Table 2 for the statistics).

**Table 2:** **Means (SD) for facial movements after the disgust stimulus, as displayed by expressers and observers**.

	Expresser	Observer	*F*(1, 54)	*p* <	${\text{η}}_{p}^{2}$
AU43 (closing eyes) + looking away	1.05 (1.18)	0.16 (0.46)	26.63	0.0001	0.33
AU9/10 (nose wrinkling)	1.36 (1.15)	0.59 (0.89)	15.96	0.0001	0.23
AU4 (frowning)	1.46 (1.19)	0.64 (0.84)	15.36	0.0001	0.22
AU12 (smiling)	1.36 (1.07)	1.63 (1.04)	2.33	0.13	0.04

**Note*. Facial actions are coded on a scale ranging from 0 (*not visible*) to 3 (*strongly visible)**.

The absence of disgust mimicry, as defined by non-significant correlations between expressers’ and observers’ facial disgust displays, may also be explained by a lack of empathy. However, the correlation between two of the three facial displays by the expresser and empathic feelings of the observer were significant: frowning (*r* = 31, *p* = 0.024), and looking away (*r* = 0.30, *p* = 0.027). Thus, the more the expresser looked away or frowned, the stronger the empathy evoked in the observer.

### Emotional mimicry in reaction to pride

In order to examine pride mimicry, we computed correlations between the smiles of observers and expressers. Across conditions, no significant correlations were found, but when computing separate correlations for intimates and strangers, we found a significant correlation for smiling for intimates (*r* = 0.59, *p* = 0.001), and not for strangers (*r* = 0.07, *p* = 0.73).

We then conducted a repeated measures ANOVA with nature of relation (intimates, strangers) as between-subjects factor, expresser and observer as within-subject factor, with the intensity of smiles (AU12) as dependent variable. We found a significant univariate effect of expresser-observer, *F*(1, 54) = 4.37, *p* = 0.041, ${\text{η}}_{p}^{2}=$ 0.07, for smiling, and no effect of the nature of the relation. Expressers (*M* = 1.88, SD = 0.69) smiled more than observers (*M* = 1.50, SD = 1.32).

### Recognition of disgust

We first examined whether observers accurately recognized the expresser’s facial display as disgust. Twenty-one percent (*N* = 12) of the observers did not recognize the expression as disgust, but as another emotion. Repeated measures ANOVA with perceived intensity of disgust, stress, irritation, and surprise (the other emotions were not observed, and therefore are not included in the analysis) and nature of relation showed a significant effect of emotion, *F*(3, 52) = 73.41, *p* < 0.0001, ${\text{η}}_{p}^{2}=$ 0.81. Disgust was perceived as more intense (*M* = 4.07, SD = 2.62) than was surprise (*M* = 1.11, SD = 1.92), irritation (*M* = 0.42, SD = 1.22), or stress (*M* = 0.05, SD = 0.29). No effect of nature of the relation, *F*(3, 52) = 0.69, *p* = 0.69, was found.

To test whether the relation between the expression and recognition of disgust was mediated by mimicry, we conducted a series of regression analyses following Baron and Kenny (1986). The first regression showed that disgust expression significantly predicted disgust recognition, *b* = 0.30, SE = 0.11; [*t*(54) = 2.77, *p* = 0.008]. Second, disgust expression did not significantly predict our proposed mediator, *b* = 0.03, SE = 0.07; [*t*(54) = 42, *p* = 0.68], and the mediator also did not predict the recognition of disgust *b* = −0.09, SE = 0.22; [*t*(54) = −0.40, *p* = 0.69], thus no support for mediation by mimicry was found.

We then conducted similar regression analyses with empathy as mediator. First, we found a significant relation between disgust expression and empathy as reported by the observer, *b* = 0.12, SE = 0.04; [*t*(54) = 2.60, *p* = 0.21]; second, empathy significantly predicted the recognition of disgust, *b* = 0.98, SE = 0.33; [*t*(54) = 2.99, *p* = 0.004]. When we added empathy to disgust expression as a predictor of recognition, empathy remained a significant predictor, *b* = 0.74, SE = 0.34; [*t*(54) = 2.18, *p* = 0.034], but the relation between disgust expression and recognition also remained significant (*b* = 0.24, SE = −0.11; [*t*(54) = 2.11, *p* = 0.04]. A Sobel test showed that the indirect path was significant, *S* = 1.97 (SE = 0.057, *p* = 0.049), showing that disgust recognition was partly mediated by empathy.

We further explored whether the relationship may also be reversed, such that the empathy that is evoked by an emotion expression is mediated by emotion recognition. Additional analyses testing this model showed that recognition of disgust significantly predicted empathy by the observer, *b* = 0.151, SE = 0.051; [*t*(54) = 2.98, *p* = 0.004], however, when adding emotion recognition to the equation, disgust expression disappeared as a significant predictor of empathy: *b* = 0.08, SE = 0.046; [*t*(54) = 1.66, *p* = 0.10]. In other words, disgust recognition fully mediated the relation between expression and empathy.

### Recognition of pride and happiness

We examined whether observers accurately interpreted the expresser’s facial display as pride. Sixty-six percent (*N* = 38) of the observers did not recognize the facial expression as pride; 32% (*N* = 19) did not even recognize it as happiness. Repeated measures ANOVA with the perceived intensity of pride, happiness, and surprise and nature of the relation as between-subjects factor showed a significant effect of the emotion, *F*(2, 54) = 13.04, *p* < 0.0001, ${\text{η}}_{p}^{2}=$ 0.33. The most intensely perceived emotion was happiness (*M* = 2.98, SD = 2.62), followed by pride (*M* = 1.59, SD = 3.00) and surprise (*M* = 0.61, SD = 2.07). Nature of the relationship was not significant, *F*(2, 54) = 0.54, *p* = 0.40. We then tested whether smiling predicted the recognition of happiness (we did not include pride as dependent measure, because pride was not recognized by 66% of the participants). The regression analysis was not significant for happiness, *b* = 0.42, SE = 0.59; [*t*(54) = 0.40, *p* = 0.49]. We further examined whether empathy was a significant predictor of happiness, and found that it was not, *b* = −0.30, SE = 0.41; [*t*(54) = −0.72, *p* = 0.48], but the interaction between empathy and nature of the relationship was marginally significant, *b* = 0.33, SE = 0.17; [*t*(54) = 1.93, *p* = 0.059], suggesting that in the intimate condition more empathy resulted in the perception of marginally more intense happiness.

## Discussion

The present study examined mimicry of disgust and pride, which were chosen because they are discrete emotions, but different from the most frequently studied emotions, namely anger and happiness. We used an interactive setting because the social implications of mimicry can best be studied in an actual social interaction. We successfully evoked disgust by having one participant smell a vile odor from a bottle, and pride by giving the participant a compliment after a seemingly difficult task. In both cases, another participant (either an intimate or a stranger) was watching and the faces of both persons were videotaped with two cameras. We assumed that both disgust and pride can signal negative social intentions, which would lead participants to refrain from mimicry. A disgust face can be easily interpreted as offensive (see Chapman et al., 2009) and the expression of pride can signal social distance (Markus and Kitayama, 1991; Fischer and Manstead, 2008).

We found support for the hypotheses that the facial display of disgust was not mimicked, whereas smiles – evoked after the disgust and the pride stimuli – were mimicked, but only among intimates. This is in line with a Social Contextual view on emotional mimicry (see Hess and Fischer, in revision), which states that facial displays of emotions are only mimicked if they serve an affiliation function. The mimicry of emotion should signal empathy or understanding and thereby foster social bonds and should be inhibited when its social consequences can be potentially negative. An affiliation context is determined by both the nature of the emotional signal and the relationship between expresser and observer. The mimicry of negative “attack” emotions (see Roseman et al., 1994) or socially distancing emotions (Markus and Kitayama, 1991) is therefore not likely in most interactive contexts, because it is less probable that these are seen as signals of support or understanding. Looking at a disgust face is somewhat threatening or at least unpleasant, even if you know that the other person is not disgusted by you.

Still, previous research has found mimicry of negative emotions, which requires an explanation. We believe that this is due to the fact that mimicry has been defined and operationalized in different ways. In the current study we operationalized mimicry as a significant correlation between facial expressions of expresser and observer within a fixed time frame, indicating that facial movements of expressers and observers occurred simultaneously within this time frame (see also Hess and Bourgeois, 2010). In research using photos or video’s, however, the mere occurrence of a congruent facial response (i.e., frowning in response to an angry face) has been operationalized as mimicry. Whereas we found no significant correlations, observers did show facial actions in reaction to the disgust face of the other participant. For example, although observers hardly closed their eyes or looked away (AU43) – which makes sense considering that this would be the immediate reaction to the vile smell that they however never directly experienced – they did sometimes frown or wrinkle their nose. In our view however, correlations between facial movements are a more adequate operationalization of mimicry, although we acknowledge that the observer’s less intense facial actions related to disgust can at least be seen as assimilative emotion displays (see also Tiedens and Fragale, 2003).

The fact that observers showed less intense emotion displays can be the result of different processes, and the present data are not conclusive in this respect. First of all, it can simply be the result of the fact that the emotional stimulus for the observer, i.e., the other person’s face, was less intense than the emotional stimulus that evoked the disgust in the expresser, namely the vile smell in the bottle. Second, it may also point to an inhibition of the mimicry reaction, which is overriden by the motivation to show concern and empathy. The observers’ facial reaction to the expressers’ disgust would then still reflect empathy, even though it did not meet our strict criteria of mimicry. This idea is supported by the positive correlations between the expresser’s display of disgust and self-reported empathic feelings on the part of the observer, but also by the mimicry of smiling after the disgust stimulus.

The finding that smiling was mimicked only in the intimate condition further supports a Social Contextual view of mimicry and is in line with previous studies (Hess and Bourgeois, 2010; Stel et al., 2010). Our participants’ mimicry of smiling clearly served an affiliation function, because they only showed smile mimicry when they were familiar with each other. These smiles therefore may reflect an empathic response in a situation where a negative emotion was evoked. These joint smiles can emphasize shared bonding, but also awkwardness or amusement about what was happening during this event. In a situation where negative emotions are elicited, observers may seek positive facial signals in order to mimic their feelings of empathy. It should be noted, however, that the fact that strangers did not show smile mimicry does not mean that strangers did not smile, as can be observed from the means. It merely indicates that they did not respond to each other’s smiles. This absence of smile mimicry in our view indicates the absence of an affiliation motive, which may be due to the fact that the context was non-affiliative (display of disgust), and the relationship was not affiliative. There may be situations where individuals still may have a motive to affiliate with the other person, however, we suggest that this is mostly the case when they share a common goal or when the situation requires affiliation. This was not the case in the present situation.

As predicted, the mimicry of smiles in reaction to the pride stimulus was also only found in the intimate condition. We argued that pride would be more easily shared with intimates than with strangers, which is supported by the fact that intimates, and not strangers, mimic each other’s smiles. It should be noted that pride (and happiness) were not reported as very intense. In addition, these feelings were not significantly correlated with the intensity of the smiles. Various studies have shown that pride has a unique expression and can be differentiated from other positive emotions (e.g., Tracy and Robins, 2004; Hawk et al., 2009; Mortillaro et al., 2012), but these studies have mostly used prototypical expressions, and predominantly bodily expressions, whereas we used spontaneous pride expressions. In real life – and in the current experiment – pride is often mixed with happiness, or surprise, and thus the expression of such a blend emotion may have been less easy to recognize. In addition, smiles have many meanings (see also LaFrance et al., 2003; Niedenthal et al., 2010), and therefore correlations with specific mental states may be hard to find. Finally, the reported feelings of pride were not very intense, and thus would not always result in a prototypical expression. In some cases, the participants did not seem proud at all, because they found it obvious to have given the correct answer to the question which of the crying babies was a boy.

This all means that the compliment may have been a more ambiguous stimulus than the repulsive smell, and this may explain why no significant determinants of pride recognition have been found. The ambiguity of the situation may also provide an alternative explanation of why smile mimicry only occurs in the intimate condition. Intimates seek support and may wish to strengthen their relationship more than strangers, especially in ambiguous situations. Uncertainty evokes the need for safety and bonding, and participants would then rely more on people they like (see e.g., Likowski et al., 2008; Stel et al., 2010), or who were supportive or rewarding in the past (see also Sims et al., 2012).

The empathy that was evoked by the expression of disgust also played a prominent role in the recognition of disgust, and to a lesser extent in the recognition of happiness (pride was recognized so badly that we did not further test factors influencing pride recognition). We examined whether mimicry and or empathy would improve the correct recognition of emotions. We found no support for this mimicry function. Instead, the more women expressed disgust, the better observers recognized disgust, and this was partially mediated by the reported empathy observers felt with the other person. In other words, the empathy felt for the other person helped observers to perceive higher levels of disgust. In addition, perceiving disgust in turn also increased empathy with the other. Empathy therefore seems to be a facilitating mental state as well as an implication of emotion recognition.

The finding that mimicry did not help to accurately recognize emotions is not inconsistent with previous studies. In studies where mimicry effects on recognition have been found, these were mostly found when mimicry was manipulated, when speed of recognition was measured, or when subtle emotion stimuli were used (e.g., Stel and Vonk, 2010; Hawk et al., 2012). In the present research, mimicry was spontaneous, and was only found for positive emotion displays and thus it is not surprising that it did not influence disgust recognition. The fact that mimicry also did not influence pride/happiness recognition may be explained by the fact that the pride expression did not result in pride recognition. Mimicry can only play a role when the emotion is clearly expressed and interpreted.

This study has limitations, which simultaneously emphasize its strengths. The fact that data were collected in a natural setting enhances the ecological validity of the study. We had participants with a variety of backgrounds, who were all very motivated to take part in the experiment. Moreover, an interactive setting as used in the current study, where two individuals see each other and react to each other, is most adequate to study social functions of emotion mimicry. In actual social interactions, social regulation processes are prompted and observers may adjust their facial reactions more or less automatically when they expect negative social consequences (see e.g., Manstead and Fischer, 2001; Evers et al., 2005). Studies of mimicry in more natural social interactions are scarce, and we think that this makes the contribution of this study valuable.

We should also acknowledge, however, that the experimental environment was not always as standardized as we would have liked. There was noise that could have distracted, and participants were not alone when completing the questionnaires. Having acknowledged these limitations, however, we think that the present results show that we do not automatically mimic negative emotions, but rather seek out the positive signals that are only mimicked when the context is affiliative. We believe that this study can potentially open up a new avenue of research on emotional mimicry in which its social implications, and thus boundary conditions, are taken into account.

## Conflict of Interest Statement

The authors declare that the research was conducted in the absence of any commercial or financial relationships that could be construed as a potential conflict of interest.

## Footnotes

^1^We also coded looking away as AU43, although this is not listed in the FACS manual. However, because looking away in this context had the same effect as closing the eyes, and almost always coincided with AU43, we found it appropriate to include this movement.

^2^Seven participants forgot to fill in this part of the questionnaire.
